# The Role of the Automated Implantable Cardioverter Defibrillator (AICD) for Secondary Prevention in the COVID-19 Era

**DOI:** 10.7759/cureus.15783

**Published:** 2021-06-20

**Authors:** Andrew V Doodnauth, Jordan I Zhou, Krunal H Patel, Fadi Yacoub, Julian Dunkley

**Affiliations:** 1 Internal Medicine, State University of New York (SUNY) Downstate Medical Center, Brooklyn, USA; 2 Internal Medicine, State University of New York (SUNY) Downstate College of Medicine, Brooklyn, USA

**Keywords:** covid-19, sudden cardiac arrest, ventricular fibrillation, sars-corona virus 2, implantable cardioverter-defibrillator

## Abstract

Life-threatening arrhythmias have been variably reported among patients hospitalized for COVID-19 infection. Sudden cardiac arrest (SCA) in COVID-19 patients is an alarming concern for clinicians. Multiple factors play an important role in the development of SCA in patients with severe systemic illness. We describe a case of COVID-19 in a New York City hospital in Spring 2020 that rapidly developed SCA and, before discharge, received a single lead transvenous implantable cardioverter defibrillator for secondary prevention. This case highlights the use of an automated implantable cardioverter-defibrillator as a secondary preventive measure irrespective of left ventricular function as a means of preventing recurrence of SCA as a sequela of COVID-19.

## Introduction

COVID-19 has now spread from Wuhan, China, across more than 200 countries and transformed into a pandemic. Sudden cardiac death (SCD) in COVID-19 patients is an alarming concern for clinicians. The incidence of SCD has been reported to be growing both in community and hospital settings [1]. Disturbingly, a recent study analyzing the data on 141 fatal cases of confirmed COVID-19 at a hospital in Wuhan, China, found sudden cardiac arrest (SCA) (n=11, 8%) to be the third-highest direct cause of death [2]. Although the direct causal association of SCD and COVID-19 remains unproven, our discussion highlights plausible mechanisms that may have led to the development of malignant arrhythmia in our patients.

An automated implantable cardioverter defibrillator (AICD) is the preferred therapeutic modality in most survivors of SCA. However, ill-defined management guidelines for patients recovered from COVID-19-associated cardiac injury and an absence of data on the long-term sequelae of COVID-19 complicates how secondary prevention of SCA and SCD should be approached. We describe a case of COVID-19 in a New York City hospital in Spring 2020 that rapidly developed SCA after presenting to the emergency room. We weigh in on the importance of AICD placement for secondary prevention, irrespective of left ventricular function in this unfathomable disease.

## Case presentation

**Figure 1:**
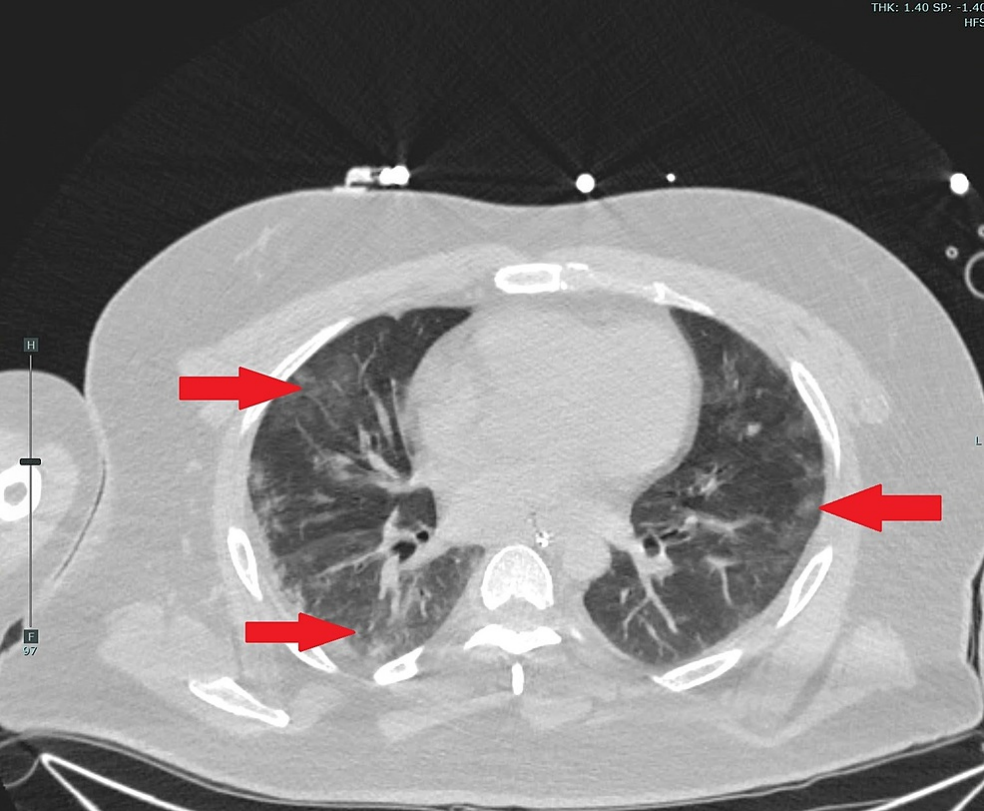
Non-contrast computed tomography chest showing bilateral ground glass opacities (arrows) suggestive of atypical pneumonia.

A 35-year-old-male with a past medical history notable for insulin-dependent diabetes mellitus presented to the emergency department with hypoxia, subsequently developing ventricular fibrillation. Advanced cardiac life support (ACLS) protocol was initiated, and after two rounds of cardiopulmonary resuscitation, we achieved the return of spontaneous circulation with successful rapid sequence intubation. Initial labs were consistent with florid diabetic ketoacidosis (DKA) and severely elevated inflammatory markers consistent with active COVID-19 infection. Non-contrast computed tomography revealed bilateral ground-glass opacities suggestive of atypical pneumonia (Figure 1) - nasopharyngeal SARS-CoV-2 PCR; positive. The patient was admitted to the medical intensive care unit for SCA secondary to presumed COVID-19 pneumonia.

**Figure 2:**
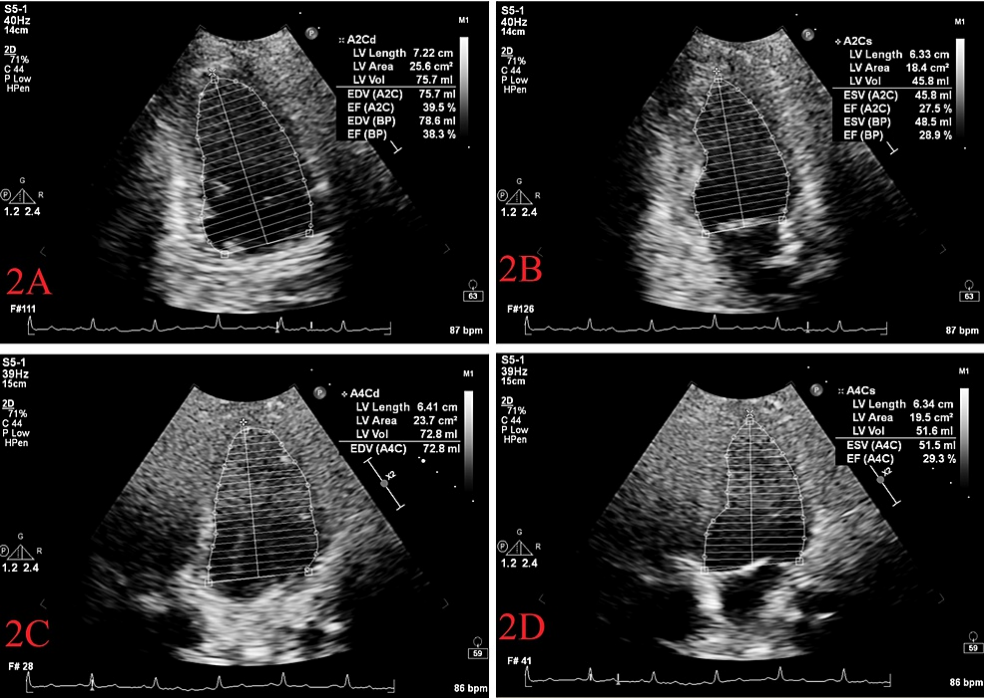
Modified Simpson method: (A) apical two chamber diastole; (B) apical two chamber systole; (C) apical four chamber diastole; (D) apical four chamber systole. A2C = Apical two chamber; A2Cd = Apical two chamber diastole; A2Cs = Apical two chamber systole; A4Cd = Apical four chamber diastole; A4Cs = Apical four chamber systole; LV = Left ventricular; BP = Biplane; EDV = End diastolic volume; EF = Ejection fraction; ESV = End systolic volume

Serial troponin-I and serial electrocardiograms were remarkable for ischemic changes. A continuous rise in cardiac biomarkers was suggestive of myocardial necrosis. Transthoracic echocardiogram (TTE) demonstrated global hypokinesis with an approximated ejection fraction of 35% (Figures 2A-2D). Cardiac catheterization was initially deferred due to hemodynamic instability. The patient was started on guideline-directed medical therapy (GDMT) for acute coronary syndrome/heart failure and treated per hospital protocol for COVID-19 infection and DKA.

**Figure 3:**
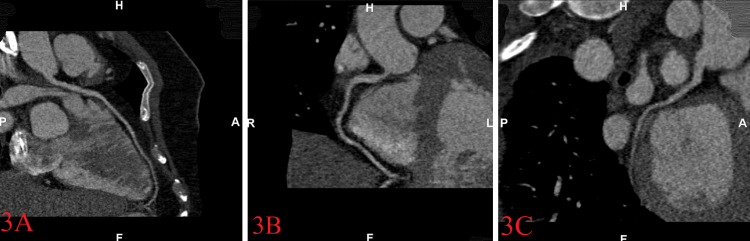
Coronary CT angiography showing the coronary arteries arise in normal positions with no evidence of coronary stenosis or plaque in the left anterior descending (A), right coronary (B), and left circumflex arteries (C). Total calcium score of zero indicating the absence of calcified plaques in the coronary tree. Labels: H = Head; F = Feet; P = Posterior; A = Anterior; R = Right; L = Left

Seven days after admission, the patient was successfully extubated and downgraded to medicine. Systemic inflammatory markers were trended daily but remained elevated although the patient was improving clinically. Two weeks later, before discharge, a coronary computed tomography angiography (CTA) was performed, showing no evidence of coronary stenosis or plaque (Figures 3A-3C). Repeat TTE revealed a significant improvement of left ventricular systolic function, an ejection fraction of 55%, no pericardial fluid, no wall motion, or valvular abnormalities. We did not pursue an electrophysiological study (EPS) due to high clinical suspicion of COVID-19-induced ventricular fibrillation.

However, we placed a transvenous single lead AICD irrespective of improved left ventricular systolic function due to concerns for recurrence of fatal ventricular arrhythmia as a sequela of COVID-19. The patient was safely discharged home with outpatient cardiology follow-up.

## Discussion

Life-threatening arrhythmias have been variably reported among patients hospitalized for COVID-19 infection. For example, a retrospective single-center case series from Wuhan, China, found that 23 (16.7%) of 138 hospitalized patients infected with COVID-19 pneumonia had arrhythmias [3]. Another study from Wuhan, China, of 187 patients with confirmed COVID-19 found that 52 patients (27.8%) had a myocardial injury resulting in cardiac dysfunction and arrhythmias, with 11 (5.9%) patients experiencing ventricular tachyarrhythmias [4].

Multiple factors play an important role in the development of SCA in patients with severe systemic illness. Factors include but are not limited to hypoxia, acidosis, dyselectrolytemia, volume imbalances, drug-drug interactions, autonomic nervous system activation, severe inflammation, ischemia, and direct viral involvement of the myocardium [1].

Underlying ischemia was thought to be the precipitant of our patient’s SCA due to the initial rhythm strip on presentation revealing ventricular fibrillation and the persistently elevated cardiac troponin. This continuous rise in cardiac troponin was of concern. A retrospective single-case series study from Wuhan, China, found that patients with elevated troponin levels had higher rates of malignant arrhythmias including ventricular tachycardia/ventricular fibrillation (11.5% vs. 5.2% of patients) [4]. Coronary CTA for our patient showed no evidence of coronary stenosis or plaque. The patient’s coronary calcium score of 0 was consistent with a type-II myocardial infarction secondary to persistent hypoxia and consequent demand-supply mismatch, resulting in myocardial injury [1].

To date, the exact mechanism has not been confirmed, but possible mechanisms by which COVID-19 may cause cardiac damage-inducing malignant arrhythmia may include inflammatory responses and cytokine storm, direct attack to cardiomyocytes, and inducing severe hypoxia [5]. Per chart review, we documented elevated systemic inflammatory markers on the initial workup, which remained persistently elevated for the duration of the hospital course.

While direct myocardial injury caused by COVID-19 and underlying cardiovascular disease is associated with arrhythmias, high-grade systemic inflammation can also play a major role in contributing to malignant arrhythmia [6]. Inflammatory cytokines such as IL-6, tumor necrosis factor (TNF) can cause “inflammatory cardiac channelopathies” by inducing gap junction dysfunction in atrial myocytes and prolonging the action potential duration and/or QT-interval through modulating cardiac ion channels such as Ca^2+^ and K^+^ [7]. In addition, these inflammatory cytokines can promote electrical instability by inducing cardiac sympathetic nervous system hyperactivation [8,9].

Before the patient’s SCA, the patient was in severe respiratory distress in transit to the emergency department found to be hypoxic with SpO_2_ of 85% on 15L of supplemental oxygen via a non-rebreather mask. The current literature supports the important electrophysiologic effects of hypoxia on the heart. In particular, hypoxia secondary to direct viral tissue involvement of lungs can lead to arrhythmias in addition to hypoxia-induced apoptosis of cardiac myocytes [8]. Second, hypoxia can activate anaerobic glycolysis causing decreased intracellular pH and increasing cytosolic calcium levels, resulting in early and late depolarization and temporal alterations in the action potential duration. Last, increased extracellular potassium levels secondary to hypoxia can decrease the threshold potential, accelerating electrical conduction [1,10].

Repeat TTE before discharge showed complete recovery of left ventricular systolic function with an ejection fraction of 55%. The comprehensive metabolic panel, complete blood count, and the patient's vitals were unremarkable. With no suspected or confirmed heritable syndromes or pertinent family history and with all potentially known reversible causes including acute myocardial infarction, transient ischemia, cardiac tamponade, anemia, hypothermia, hypovolemia, pulmonary embolism, tension pneumothorax, electrolyte imbalance, antiarrhythmic drugs with proarrhythmic effect, hypoxia, or sepsis [11] ruled out, the inpatient medical team felt comfortable discharging the patient on GDMT for new-onset heart failure with outpatient cardiology follow-up. However, because there was high suspicion for COVID-19 as the culprit, the electrophysiology team had concerns for the development of future episodes of malignant arrhythmias due to the unknown pathophysiology, raising the question of secondary prevention.

In the remodeling stage of inflammation, variable degrees of myocardial scarring allow for arrhythmia development [1,12]. Multiple studies have shown the short-term cardiovascular effects within months after recovery from COVID-19. A cohort study including 100 patients who recently recovered from COVID-19, cardiovascular magnetic resonance (CMR), was conducted 71 days after initial diagnosis. This revealed cardiac involvement in 78 patients (78%) and ongoing myocardial inflammation in 60 patients (60%) with associated myocardial edema, small pericardial effusion, and focal linear subepicardial and patchy mid-wall late gadolinium enhancement (LGE). These results were found independent of preexisting conditions, severity and overall course of the acute illness, and time from the original diagnosis [13].

The short-term cardiovascular effects of COVID-19 remain a challenge when considering secondary management. Still, the lack of data available for long-term COVID-19 cardiovascular complications further complicates this challenge.

Due to the constraints of the pandemic, we were unable to obtain an endomyocardial biopsy with histopathologic examination to diagnose viral myocarditis definitively. However, our clinical findings of fatal ventricular arrhythmia, new-onset heart failure symptoms, and TTE demonstrating global hypokinesis allowed for a clinical diagnosis of acute viral myocarditis, consistent with the 2013 European Society of Cardiology position statement on myocarditis [14].

Despite the lack of data available for long-term COVID-19 cardiovascular complications, a long-term study evaluating outcomes in 466 patients with biopsy-proven inflammatory cardiomyopathy from other viral pathogens was conducted after a mean follow-up of 43.4 months. This study revealed that four (0.9%) patients experienced SCD, 15 (3.2%) patients experienced aborted SCD due to resuscitation, and 12 (2.56%) patients experienced aborted SCD due to ICD shock. Increased risk of adverse events during follow-up was associated with reduced left ventricular ejection fraction and resuscitation before admission [15,16]. These factors are of particular concern as our patient presented with an ejection fraction of 35% and required ACLS secondary to ventricular fibrillation. Another study following 82 patients that were predominantly young males with endomyocardial biopsy-proven active myocarditis at a mean follow-up of 147 months found that, of the 23 (28%) of patients that died, sudden cardiac death (SCD) (3; 13%) was the second most prevalent cause of death [16,17]. The high long-term rates of SCA and aborted SCA in biopsy-proven myocarditis patients raise the concern of cardiomyopathy after the resolution of viral myocarditis secondary to SARS-CoV-2.

The question remains whether patients with improved left ventricular systolic function following resolution of viral myocarditis are also susceptible to SCA or SCD. A long-term study that evaluated the rates of ventricular arrhythmias in patients with myocarditis at different stages found an increased prevalence of ventricular arrhythmia in patients with healed myocarditis, even if ejection fraction is >50% [16,18]. In light of other viruses causing the increased prevalence of arrhythmias despite ejection fraction >50% and possible SCD during the convalescent phase of viral myocarditis, it is reasonable to presume that survivors of SCA due to COVID-19 with no structural heart disease and an ejection fraction of >50%, such as with our patient, are susceptible to repeat cardiac arrhythmias.

Even as we have taken giant strides since the beginning of 2020 in learning about COVID-19, many unknowns remain. Despite ostensibly recovered cardiac function in our patient, the electrophysiology team erred on the side of caution, feeling that there would be a risk for a repeated episode of fatal ventricular arrhythmia, and placed an AICD for secondary prevention. Keeping this high index of suspicion in survivors of SCA secondary to COVID-19, even if cardiac function is ostensibly recovered, can help prevent the recurrence of SCA or SCD.

## Conclusions

In conclusion, the exact long-term risk of arrhythmia for patients who recover from the acute phase of COVID-19-associated cardiomyopathy is unclear. Nonetheless, our discussion emphasizes the growing data on subclinical cardiac injury in COVID-19 patients even after recovery. This case highlights the use of an AICD as a secondary preventive measure irrespective of left ventricular function as a means of preventing recurrence of SCA and SCD as a sequela of COVID-19.
